# The Underlying Mechanisms and Role of Negative Pressure Wound Therapy in Chronic Diabetic Wound Healing: A Systematic Review and Meta‐Analysis

**DOI:** 10.1111/iwj.70961

**Published:** 2026-06-01

**Authors:** Roxana Moscalu, Mihaela Moscalu, Adam J. Reid, Marco Domingos, Adam Stevens, Jason K. F. Wong

**Affiliations:** ^1^ Blond McIndoe Laboratories, Division of Cell Matrix Biology and Regenerative Medicine, School of Biological Sciences, Faculty of Biology, Medicine and Health The University of Manchester, Manchester Academic Health Science Centre Manchester UK; ^2^ Department of Preventive Medicine and Interdisciplinarity, Faculty of Medicine Grigore T. Popa University of Medicine and Pharmacy Iassy Romania; ^3^ Department of Plastic Surgery & Burns, Wythenshawe Hospital Manchester University NHS Foundation Trust, Manchester Academic Health Science Centre Manchester UK; ^4^ School of Mechanical, Aerospace and Civil Engineering The University of Manchester Manchester UK; ^5^ Tommy's Maternal and Fetal Health Research Centre, Faculty of Biology, Medicine & Health, 5th Floor St. Mary's Hospital The University of Manchester Manchester UK

**Keywords:** chronic wound healing, diabetes, diabetic foot ulcers, NPWT

## Abstract

Although Negative Pressure Wound Therapy (NPWT) has been increasingly used in wound care to improve impaired healing, there is little scientific evidence supporting its role and underlying biomolecular mechanisms. Aims of the present study are to provide a quantitative analysis of recent literature investigating NPWT in diabetic wound healing focusing on healing duration, wound closure, hospitalisation period and complications, and qualitative insight into studies analysing biomolecular mechanisms. The systematic review and meta‐analysis were conducted following PRISMA guidelines (PROSPERO: CRD42024524813). 21 studies published in PubMed, Cochrane Library, EMBASE between 2019 and 2024 were included. Clinical studies indicated NPWT was superior to standard care dressings (SCD), promoting faster wound healing with significantly reduced hospitalisation times by 7.8 days (95% CI: −14.2 to −1.4, *p* = 0.017), and significantly reduced complications rates, particularly major and minor amputations (95% CI: −10.2 to −1.3, *p* = 0.01). Mechanistic in vitro and animal studies highlighted NPWT can reduce local inflammation, oxidative stress, support angiogenesis and improve scarring, essential components of normal healing. Although studies suggest NPWT is more effective than SCD for diabetic wound healing, the paucity of studies, small cohorts and scarce outcomes consistency make defining clear conclusions challenging. There is still more evidence required to fully understand NPWT's role in the complex diabetic wound healing.

## Highlights

- NPWT can help reduce diabetic wound healing times and patient hospitalisation time when compared to standard care dressings.
- Across literature, NPWT was found to significantly reduce risk of major and minor amputations.
- In vitro and in vivo studies indicated NPWT can support angiogenesis and reduce local inflammation and oxidative stress and improve scarring, mechanisms associated with normal healing pathways.
- Future studies should report more standardise analysis timepoints and outcome measures and incorporate deeper molecular investigations to better understand the mechanisms of wound healing, chronicity and role of NPWT in diabetic.

**Abbreviations**
ANG2: angiopoietin‐2
DFU: diabetic foot ulcers
DM: Diabetes mellitus
En1: engrailed‐1
ENF: En1 linage‐negative fibroblast
NPWT: negative pressure wound therapy
SCD: standard care dressings
SMWC: standard moist wound care
s‐NPWT: single use NPWT
SYRCLE: Systematic review centre for laboratory animal experimentation
t‐NPWT: traditional NPWT
t‐PRDX2: tissue margin peroxiredoxin‐2
YAP: yes‐associated protein

## 1 Introduction

Complex, non‐healing diabetic wounds have long had a considerable socioeconomic impact on public healthcare systems globally. In 2024, it was estimated that over 588.7 million people aged 20–79 years were affected by diabetes mellitus (DM), with the expectation that numbers will increase exponentially by 2050 [1]. In UK, the latest recorded prevalence of diabetes in the adult population was 9.2% [1]. The same report from 2024 attributed a socio‐economic burden of over USD 23 billion to DM‐related costings in the UK alone [1], while a previous study from 2019 on the costs of diabetic foot ulcers (DFU) on the National Health Service (NHS) noted that over £900 million annually have been attributed to the management of DM‐related wounds, equivalent of approximately 1% of England's yearly NHS budget [2]. These costs are mainly linked to the long‐term and complex multidisciplinary care of ulcers associated with the disease.

The development of diabetic ulcers mainly on the pressure loading areas, or bony prominences of the feet and legs is becoming an increasingly common problem among DM patients, requiring complex interventions and management techniques for prolonged periods of time.

The development of diabetic ulcers occurs predominantly in the lower limbs and requires complex interventions and wound management techniques for prolonged periods until healing is achieved. This is a significant challenge for healthcare systems globally that are experiencing an increasing number of diabetic wounds secondary to poorly implemented preventative measures [3]. This is also a major challenge for diabetic patients, as a leading cause of limb amputation (50%–70% of total limb amputations are due to DM‐related complications) and high mortality rates [2, 3].

Negative pressure wound therapy (NPWT) has been recently gaining increased interest in the management of complex wounds of various aetiologies, particularly challenging wounds such as non‐healing DFU.

NPWT has been used across a wide aetiology of wounds, including acute and subacute, traumatic, chronic wounds and ulcers (diabetic, pressure or venous ulcers), partial‐thickness burns, flaps and skin grafts as well as dehisced wounds [4]. Unlike many standard dressings or advanced wound‐care approaches that primarily address individual aspects of wound management, NPWT provides a more comprehensive therapeutic effect through four main mechanisms, including macrodeformation which facilitates wound edge contracture, thus reducing wound size, microdeformation leading to upregulation of mechanotransduction pathways, effective exudate management and control of wound microenvironment [5, 6]. These contribute to wound protection, granulation tissue formation and angiogenesis, reduction in localised oedema, and lower the risk of infections [7]. In the context of diabetic foot ulcers, this adjunctive role is reflected in the most recent International Working Group on the Diabetic Foot (IWGDF) guidelines [8] which recommend considering NPWT as an adjunct to standard care in the management of post‐surgical DFU.

Although used routinely in management of both acute and chronic wounds, its effects over biological mechanisms that promote wound healing are yet to be fully understood and explained.

The main aim of this systematic review is to provide a qualitative and quantitative analysis of the most recent studies looking into the role of NPWT in diabetic wounds healing published in the past 5 years. The hypothesis is that for the adult population with diabetic ulcers, treatment with NPWT leads to faster and more effective wound healing trajectory than standard‐of‐care dressings alone. Moreover, preclinical studies also further support the mechanistic hypothesis, suggesting NPWT could positively influence wound repair through local biomechanical and microenvironmental changes, particularly related to fibrosis and angiogenesis. Thus, the analysis will include studies comparing the effectiveness of NPWT in with current standard of care dressings for diabetic wound healing, as well as a more qualitative analysis looking into different strategies and variants of NPWT, and research studies trying to highlight the underlying mechanisms of healing promoted by NPWT devices.

## 2 Methods

### 2.1 Search Strategy

A thorough literature search focusing on the articles published in the last 5 years, to reflect most current practices and recent technologies and wound‐care pathways available in the field. This focused on complex diabetic wounds and their underlying impaired healing mechanisms. Thus, relevant research studies published between January 2019 and May 2024 were identified by two independent reviewers through 3 scholarly databases: PubMed, Cochrane Library and EMBASE. To refine our search, the following string including keywords and Boolean operators were used across all databases search engines: ((diabetic wound) OR (diabetic ulcer) OR (diabetic foot)) AND ((negative pressure wound therapy) OR NPWT OR PICO OR VAC OR (vacuum assisted closure) OR (vacuum assisted dressing) OR (topical negative pressure)).

### 2.2 Inclusion and Exclusion Criteria

This systematic review was conducted and presented following the Preferred Reporting Items for Systematic Reviews and Meta‐Analysis (PRISMA) guidelines. Its protocol was registered in the International Prospective Register of Systematic Reviews (PROSPERO, CRD42024524813). Our research questions follow the PICO methodology, as seen in Table 1.

**TABLE 1:** Study description according to the PICO (Population, Intervention, Comparison and Outcome) strategy.

Population	Description	Exclusion criteria
	Patient of any age with non‐healing diabetic wounds	Patient with non‐diabetic wounds
Intervention	NPWT alone (to include all variations of devices such as traditional NPWT, VAC, PICOTM or NPWT with irrigation)	NPWT used in combination with other therapies
Comparison	Standard care dressings or an alternative NPWT device	N/A
Outcomes	Effects/Efficacy (time to complete wound closure, wound area reduction, in hospital stay duration, complications) of NPWT in the healing process of diabetic wounds Analysis of underlying pathways or changes determined by NPWT in diabetic wound healing	Economic or costs analysis Studies that were not focused on effects of NPWT on the healing process in diabetic wounds
Study design	Randomised Control Trials Retrospective cohort studies Retrospective case–control studies In vivo studies	Clinical trials proposals Case studies/series Review articles, systematic review or meta‐analysis Letters to editor Preliminary RCT results
Full article accessible	Full text available	Full text inaccessible

Thus, the generated studies were closely reviewed for relevance; the PICO strategy is defined in Table 1. It is to note that Cochrane Library and EMBASE databases generated several entries where the full study was not available, therefore they were excluded from the systematic review. Studies in which NPWT was combined with other therapies were excluded to isolate the effects of the device alone. Likewise, economic and cost analyses were omitted so we could focus solely on NPWT's clinical and biomolecular outcomes.

### 2.3 Search Refinement

An initial search was conducted by the 2 reviewers independently in the 3 databases using the string mentioned above, then further filtered for “Full text” and “Free full text” availability and period of interest between January 2019 and May 2024. After the initial search conducted as above, a few further keywords were used for a more target filtering of articles: ‘non‐insulin dependent diabetes mellitus’, ‘diabetes mellitus’, ‘wound dressing’, ‘vacuum assisted closure device’, ‘polyurethane foam dressing’, ‘foam dressing’ and ‘PICO’. Each study was then assessed following the inclusion and exclusion criteria defined; if case of any disagreements, the reviewers would re‐analyse the studies together closely following the defined criteria until a consensus was reached. Any duplicate articles identified among the 3 databases would also be accounted in our study only once.

### 2.4 Risk of Bias Assessment

Risk of bias for each study was assessed using the relevant tools by two independent reviewers. Thus, the risk of bias was analysed using the Cochrane Risk of Bias 2 tool [9] for RCTs, Cochrane ROBINS‐I v2 tool [10] for non‐randomised case–control studies, and Systematic Review Centre for Laboratory Animal Experimentation (SYRCLE) Risk of Bias tool [11] for in vivo studies. Decision discrepancies were resolved through assessors' discussion.

### 2.5 Statistical Analysis

The meta‐analysis was performed using SPSS v.29 (IBM Ireland Product Distribution Limited, Dublin, Ireland). The model utilised standardised effect sizes corresponding to each analysed group, applying a Random‐effects model. Heterogeneity was assessed by Cochran's Q test based on a Chi‐square distribution. The subsequent *p* value was calculated. Furthermore, I^2^ percentage was calculated to assess the ratio between true heterogeneity and total variation. The extent of variation among the effects observed among different publications was referred to as tau‐squared (τ^2^). Therefore, the amount of true heterogeneity exceeding sampling biases can be assessed. The cut‐off standard value for significance was 0.05 (two tailed). The unit of analysis was the difference in means or proportions between the comparison groups. A negative effect size indicated better results in the study (intervention) group.

## 3 Results

An overview of data published between January 2019 and May 2024 on NPWT for diabetic wound healing generated 21 articles that met our inclusion and exclusion criteria and were included in the present systematic review. The outcomes of these studies will be discussed later on to analyse the breakthrough in understanding the efficiency of NPWT in diabetic wound healing, and the underlying mechanisms that can support their significant impact for promoting heal in such complex conditions.

### 3.1 Search Outcomes

**FIGURE 1:**
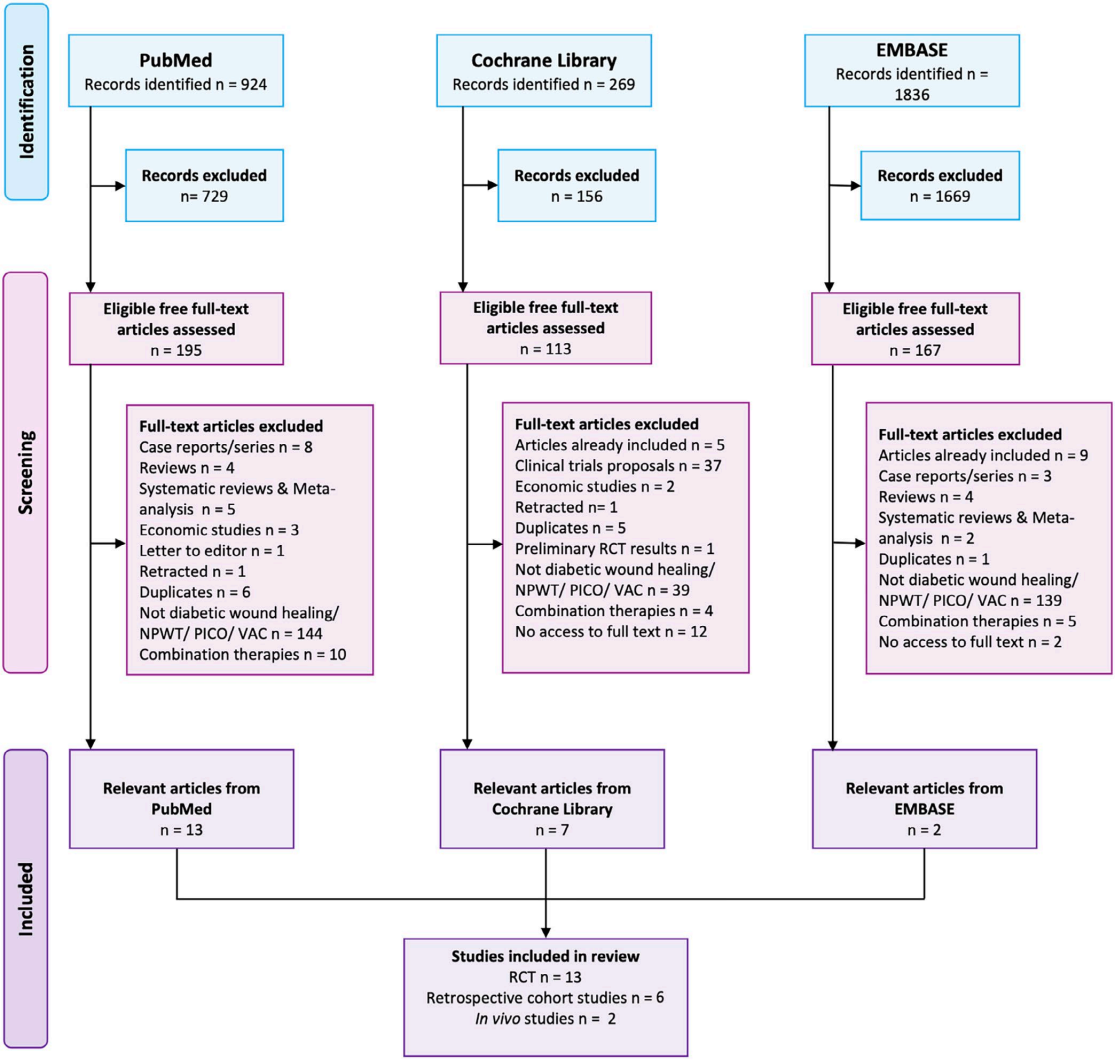
PRISM checklist for article selection and study design protocol.

The PRISMA flow diagram has been completed as shown in Figure 1 to illustrate the literature search results and our screening process. The initial search on the PubMED database generated a total of 924 results which were further reduced to only 195 potential articles after filters for “Full text” and “Free full text” availability and period of interest set to January 2019–May 2024. Similarly, total results generated on the Cochrane Library resulted in 269 entries, brought down to only 113 for the past 5 years timeline. For EMBASE, the total of 1836 articles were refined to only 167 studies of potential interest after adjusted for the last 5 year interval.

### 3.2 Study Overview

An overview of the 21 studies included is presented in Table 2. In total, the systematic review included 13 Randomised control trials (RCT), 6 retrospective cohort studies and 2 in vivo studies.

**TABLE 2:** Overview of the articles included in the study.

Author	Study design	Location and study period	Duration of observations	Sample size (*N*)	Aims	Outcomes	NWPT type	Relevant outcomes
Maranna et al. [12]	RCT	India single centre	3 months	45	Comparative DFU healing following NPWT vs. saline dressings	Granulation tissue formation, wound size reduction, hospital stay complete healing durations	VAC (KCI USA Inc) (−125 mmHg)	NPWT group presented significantly higher levels of granulation tissue, reduction in wound size, healing and hospitalisation time
Seidel et al. [13]	RCT	Germany Multicentre December 2011–August 2014	16 weeks/6 months	345	Effectiveness and safety of NPWT in DFU vs. SMWC	Primary: rate of wound closure Secondary: wound and treatment related adverse effects, amputations, wound bed preparation time, wound tissue composition, pain and patient's quality of life at 16 weeks; recurrence and wound closure at 6 months	VAC (KCI USA Inc), RENASYS (Smith&Nephew)	No significant differences in healing time or wound closure rates
Seidel et al. [14]	RCT	Germany Multicentre December 2011–October 2014	16 weeks	368	Resource utilisation for DFU treated with NPWT vs. SMWC	Resource allocation, treatment duration, length of hospital stays, rate of re‐hospitalisation, time spent on wound treatment and supportive care, dressing changes and rate of wound closure	KCI‐Granufoam black dressings and KCI‐Acti V.A.C (KCI USA Inc)	NPWT group presented significantly lower treatment duration than SMWC
Alamdari et al. [15]	RCT	Iran Single centre January 2017–December 2018	Until complete closure	60	Efficiency of NPWT in DFU healing vs. silver sulfadiazine dressings	Healing rate, size changes, rate of minor and major amputations, disability period and complications	VAC (Vacuumed V.A.C., FAPSCO, Iran) (−75 mmHg‐ −100 mmHg)	NPWH group had a significantly increased healing rate and significant reduction in wound size, number of amputations and disability duration compared to the standard care group
Anjum et al. [16]	RCT	Pakistan Single centre 6 months	8 weeks+2 weeks post wound closure	40	Efficiency of VAC dressing vs. conventional wound dressing (saline‐soaked gauze) in DFU healing	Time to healing and granulation tissue formation	VAC (−80 mmHg −120 mmHg)	VAC‐treated wounds healed significantly faster than controls
James et al. [17]	RCT	India single centre 2 years	Until complete healing	54	Efficiency of VAC in DFU wound healing vs. conventional dressings	Primary: Time to healing Secondary granulation tissue formation, complications rate	VAC (−125 mmHg)	VAC group healed significantly faster, rate of granulation tissue formation was significantly increased and significantly less pain noted
Subbiah et al. [18]	Retrospective case–control study	India Single centre January 2022–November 2022	Until discharge	50	Efficacy of VAC therapy on diabetic wound healing vs. conventional dressings	Length of hospital stay, granulation tissue formation, pain, amputation rates, wound cultures	VAC (−150 mmHg)	VAC group had significantly reduced hospital stay, lower rates of positive wound cultures, pain and amputation rates; granulation tissue formation was significantly higher than in control group
Srivastava et al. [19]	RCT	India Single centre July 2018–December 2019	3 weeks/until ulcer healed	55	Effects of NPWT in DFU healing vs. conventional dressings	Wound cultures, size, granulation tissue formation, pain, limb and wound margin temperature	NPWT (−125 mmHg)	NPWT group had significant improvements in ulcer size and granulation tissue formation, significantly more negative cultures found compared to conventional dressings. Patients reported significantly less pain with NPWT
Al‐Sabbagh et al. [20]	RCT	Single centre July–December 2018	Until complete healing (or until endpoints: amputation/death)	175	Effect of standard (−120 mmHg) and high (−160 mmHg) negative pressure on DFU healing	Rate of completely healed ulcers, time to healing, rate of major amputations and mortality	RENASYS (Smith&Nephew) (−120 mmHg vs. −160 mmHg)	High NPWT group showed a significant increase in completely healed wounds, significantly lower healing duration/amputations rates/death rates than standard NPWT
Campitiello et al. [21]	RCT	Italy Single centre September 2017–January 2018	3 weeks	59	Benefits and complication rates of standard NPWT vs. NPWT+ for DFU	Primary: healing duration and rates of complete wound closure Secondary: rates of major amputations and infections	VAC (KCI USA Inc.) (−125 mmHg)	There was a significant reduction in healing time and improvement in complete healing rates in NPWT+ group
Kirsner et al. [22],	RCT	USA & Canada Multicentre	12 weeks	156	Rate of wound closure in diabetic lower extremity ulcers between single use NPWT (s‐NPWT) and traditional NPWT (t‐NPWT)	Rate of wound closure	PICO (Smith+Nephew, Fort Worth, Texas) (−80 mmHg) as s‐NPWT vs. t‐NPWT from various manufacturers	s‐NPWT (PICO) group presented a significantly increased rate of wound closure compared to t‐NPWT
Davis et al. [23]	RCT	USA Single centre April 2016–January 2018	12 weeks	90	Efficiency of NPWT with and without irrigation on DFU healing (with infection)	Primary: healed wounds proportion after 12 weeks. Secondary: surgical wound closure, number of surgeries, length of stay, and time to healing	NPWT‐K (KCI, USA Inc), NPWT‐C and NPWT‐I (Cardinal, Dublin, OH) (−125 mmHg)	No differences in outcomes noted between NPWT‐I, NPWT‐C, and NPWT‐K for proportion of healed wounds, surgical wound closure, number of surgeries, length of stay, time to wound healing
Wu et al. [24]	RCT	China September 2022–March 2023	Until granulation tissue formation	100	Efficacy of NPWT in wound bed preparation for skin grafting in DFU vs. alginate dressings	Primary: time to surgery Secondary: skin graft survival rate, wound blood perfusion, NETs formation, polarisation of M1 and M2 macrophages	VAC (KCI USA Inc) (−125 mmHg)	NPWT group showed less time to surgery than control group, a significantly increased graft survival rate and blood perfusion. NPWT group had decreased NET formation. Macrophage polarisation from M1 to M2 phenotype was also noted with NPWT
Hohendorff et al. [25]	Retrospective case–control study	Poland	8+/−1 day	69	Effects of NPWT on plasma levels of angiopoietin‐2 (Ang2), Tie2 and microvesicles in DFU vs. standard therapy	Ang2, Tie2 and microvesicles levels	NPWT	NPWT group showed decrease in Ang2 levels by day 8
Wang et al. [26]	RCT	China July 2017–April 2018	1 week	26	Effect of NPWT on regulation mechanism of MAPK‐JNK signalling pathway vs. gauze dressings	Skin sample harvested for histological analysis and immunofluorescence analysis for iNOS, IL‐6, TNF‐α, JNK, ERK1/2 and p38 expression	NPWT (VSD Medical Science and Technology Co. Ltd., China) (−125 mmHg)	IL‐6, TNF‐α, JNK were significantly decreased in NPWT group
Liu et al. [27]	Retrospective case–control study	China January 2017–December 2019	1 week	172	Demonstrate that NPWT promotes proliferation and migration of keratinocytes by has‐miR‐203 downregulation when compared to routine dressing therapy or non‐diabetic wounds	Has‐miR‐203 levels in plasma and wound margins, and in vitro study of human keratinocytes proliferation and migration	VAC (KCI USA Inc., US) (−125 mmHg)	NPWT group significantly lower levels of has‐miR‐203 in plasma and wound margins after 1 week therapy compared to pre‐NPWT In vitro tests showed lowering levels of has‐miR‐203 during wound healing
Jia et al. [28]	Retrospective case–control study	China	1 week	3	Understand role of NPWT in DFU healing by analysing granulation tissue protein expression profiles	Granulation tissue protein profiles proteomic analysis	VAC (KCI USA Inc) (−125 mmHg)	36 proteins with significant differences identified between pre‐ and post‐NPWT samples
Tang et al. [29]	Retrospective case–control study	China	1 week	28	Changes in PRDX2 expression before and after NPWT vs. standard wound care and understand its role in DFU healing	PRDX2 expression	VAC (KCI USA Inc) (−125 mmHg)	NPWT had higher PRDX2 expression after 1 week and lower inflammatory markers. PRDX2 in wound margins could potentially be associated with healing effect of NPWT
Ludwig‐Slomczynska et al. [30]	Retrospective case–control study	Poland	8+/−1 day	36	Effect of NPWT on DNA methylation in DFU vs. standard therapy	Genome‐wide DNA analysis	RENASYS TM NPWT system (Smith&Nephew) (−120 mmHg)	NPWT group presented 426 differentially methylated regions (4 linked with complement system activation), none were found in standard treatment group
Wu et al. [31]	In vivo study	USA	10 days	20	Effects of micromechanical forces from NPWT on fibrosis pathophysiology in diabetic mice	Scarring pathway physiology factors (YAP, pro‐fibrotic fibroblasts En1), adenosine deaminase complexing protein 2 CD26 and α‐SMA	VAC (KCI USA Inc) (−125 mmHg)	NPWT presented significantly higher levels of YAP, but significantly decreased pro‐fibrotic factors (Vimentin, α‐SMA, HSP47). Fibronectin was significantly higher in NPWT group, while collagen deposition was markedly lower
Zhang et al. [32]	In vivo study	China	9 days	30	Effect of NPWT on microRNA‐126 expression vs. moist gauze dressings in diabetic mice	MicroRNA‐126 expression in wound and plasma	(VSD Medical Technology Inc., China) (−125 mmHg)	NPWT group showed significant upregulation of microRNA‐126 and VEGF compared to control group

The efficacy of NPWT devices compared with standard care dressings was identified in 8 studies, 7 RCT and 1 retrospective cohort study by Maranna et al. [12], Seidel et al. (2020) [13], Seidel et al. (2022) [14], Alamdari et al. [15], Anjum et al. [16], James et al. [17], Subbiah et al. [18] and Srivastava et al. [19]. These compared the rate of wound healing and granulation tissue formation, time to heal or duration of hospital stay and rate of complications such as amputation between the present intervention and a control (conventional dressing, saline soaked dressings, standard moist wound care (SMWC), silver sulfadiazine ointment dressings).

A comparative analysis between NPWT devices was performed by Kirsner et al. [22], who looked at the differences in outcomes between PICO (Smith&Nephew Medical Ltd., Hull, UK) and traditional NPWT devices, and Davis et al. [23] where NPWT with or without simultaneous irrigation were compared. Al‐Sabbah et al. [20] analysed the effects of different levels of negative pressure produced by the NPWT system, looking into the rate of wound healing.

Furthermore, Campitiello et al. [21] provided a unique perspective by investigating the effects of NPWT dressing wrapped around the whole affected foot rather than the conventional way of localising it to the target therapy area. Wound healing‐related pathways linked with angiogenesis, inflammation, macrophage proliferation or oxidative stress reduction in the tissue at a micromolecular level were also studied by Wang et al. [26], Liu et al. [27], Wu et al. [24], Tang et al. [29], and Hohendorff et al. [25].

Genomic and proteomic studies were performed for a more detailed insight into diabetic wound healing by Jia et al. [28] and Ludwig‐Slomczynska et al. [30] to look into the protein and DNA methylation changes influenced by traditional NPWT devices such as VAC (KCI USA Inc., US) or RENASYS NPWT system (Smith&Nephew). Zhang et al. [32] and Wu et al. (2022) [31] conducted in vivo animal studies based on diabetic mice models to analyse the effects of NPWT on various molecular factors, such as microRNA‐126 expression or pathways linked with diabetic wound healing.

### 3.3 Risk of Bias

**FIGURE 2:**
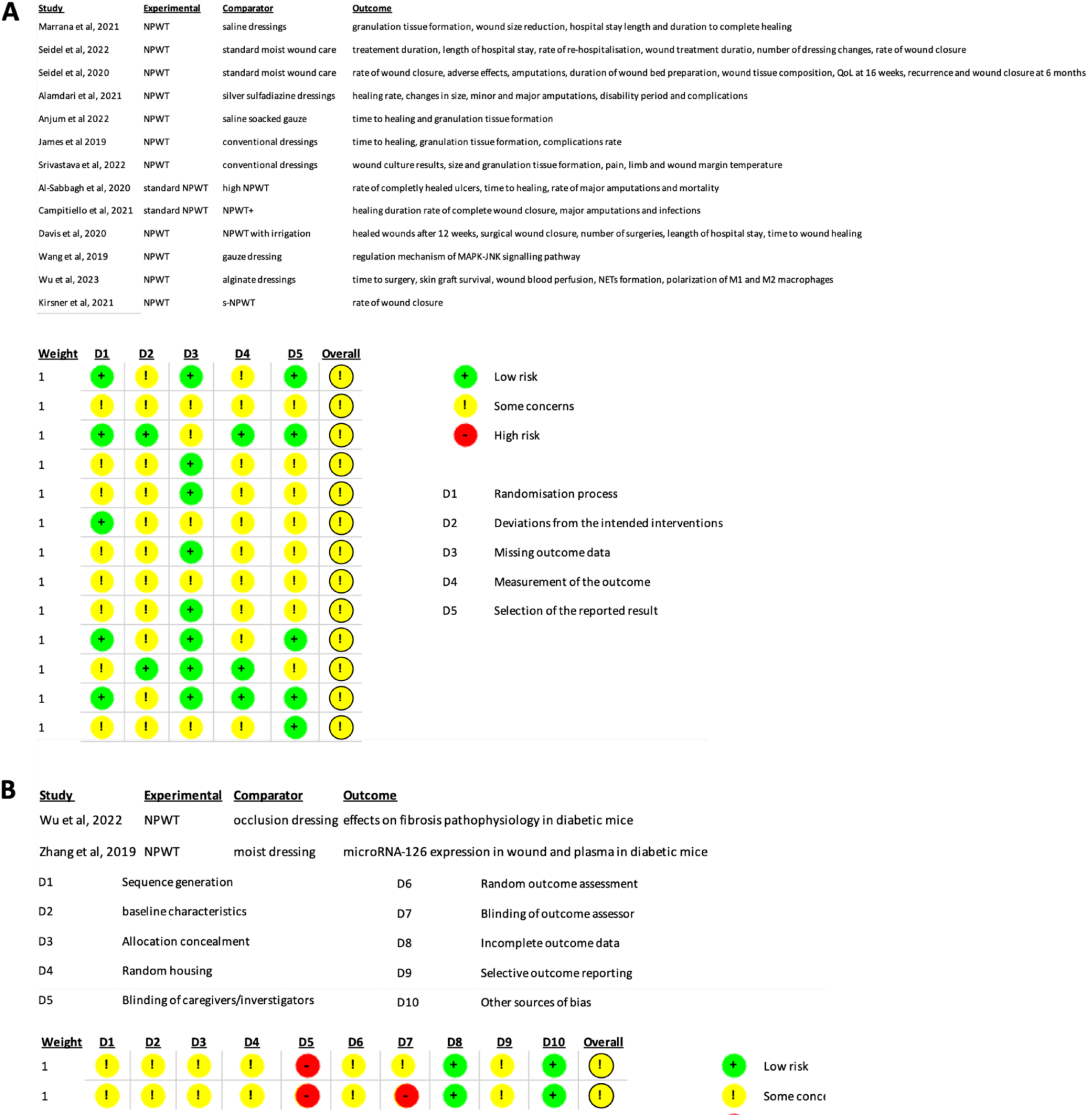
Risk of Bias for RCTs and in vivo studies. (A) Traffic light plot for risk of bias assessment of RCTs (B) SYRCLE tool risk of bias assessment for in vivo studies.

In the risk of bias assessment of the 13 RCTs included, all studies presented “some concerns” (Figure 2A). The weakest domain was the randomisation process as only one trial (Wu et al. [24]) provided full details of the sequence of generation and allocation concealment. Every study was open‐label, creating “some concerns” for deviations from intended interventions. For most studies outcome assessors were unblinded, thus raising detection bias in wound‐healing measurements. Finally, only a minority of trials cited a prospectively registered protocol, therefore selective reporting remains possible. Attrition was generally low, with only two trials (Al‐Sabbagh et al. [20] and James et al. [17]) showing notable losses, but gaps in blinding and trial registration temper out confidence in the precise magnitude of effect.

Across the 6 non‐randomised studies assessed using the ROBINS‐I tool, every investigation carried an overall judgement of “Serious” risk of bias. The predominant weakness was confounding, as none of the studies used random allocation and treatment assignment frequently depended on patient or clinician factors that were not adjusted for. Participants selection was also a concern in most studies as enrolment was often retrospective or conditional on clinical criteria, raising possibility of selection bias. In contrast, classification of interventions and adherence to treatment protocols were almost universally low risk, and missing data was minimal, with complete follow‐up reported. Outcome measures, whether objective laboratory assays or standardised wound assessments, were generally robust, but absence of any prospectively registered protocols meant that selective reporting could not be ruled out.

The studies by Wu et al. [31] and Zhang et al. [32] were assessed using the SYRCLE tool were both judged with overall “some concerns” of bias (Figure 2B). Key limitations included unclear sequence generation and allocation concealment, non‐random housing, lack of blinding for caregivers and outcome assessors. All animals completed the protocols and laboratory measurements (histology, PCR, Western blot etc.) were objective.

### 3.4 Quantitative Analysis of Clinical Studies Comparing NPWT to Conventional Dressings

Only 8 studies directly compared NPWT devices with current conventional dressings for diabetic wound healing. The cohorts comprised of 219 women, 106 in the intervention groups and 113 in the control groups, and 584 men, 256 in the intervention groups and 328 as controls. All studies used traditional NPWT devices (t‐NPWT), majority focusing on the VAC (KCI USA Inc., US). Regarding conventional dressings, SMWC or, more specifically saline soaked gauze, was the main control for the studies; only one study used silver sulfadiazine ointment dressings for the comparative analysis (Alamdari et al. [15]).

Different t‐NPWT devices were used across sites with a negative pressure ranging between −75 mmHg and −150 mmHg. Seven out of 8 studies showed NPWT is superior to conventional dressings, promoting significantly faster wound healing [12, 14, 16, 17], higher rates of granulation tissue formation [12, 17, 18, 19], achieved higher rates of complete wound closure [15] or more significant reduction in wound size [12, 15, 19], and decreased length of hospital stay [12, 18]. T‐NPWT also significantly decreased risk of complications including amputations [15, 18], caused considerably less pain [17, 18, 19], thereby improving patients' overall experience. A single multicentre study by Seidel et al. [13] was not in complete accordance with these studies; although it highlights NPWT's significant positive role in wound bed preparation, overall, it did not find NPWT superior to their standard care dressings.

NPWT's significant role in reducing rate of wound infections has also been noted, Srivastava et al. [19] and Subbiah et al. [18] illustrating a significant reduction in positive bacterial cultures for patients with diabetic wounds receiving NPWT compared to the conventional dressings.

#### 3.4.1 Outcomes of NPWT vs. Conventional Dressings Studies

Out of the 8 studies, 3 noted the difference in hospital stay between intervention and control groups. Maranna et al. [12] included 22 patients in NPWT intervention group (16 males and 6 females) with mean age of 50.2 ± 10.5 years, and 23 patients in the control group (17 males and 6 females) with mean age of 49 ± 10.1 years. The study compared t‐NPWT device VAC (KCI USA Inc., US) with saline dressings and showed a significant decrease in wound size at day 14 (40.8 vs. 21.2%, *p* = 0.008) in the intervention group compared with control, and significantly more granulation tissue formation (91.1 vs. 52.6%, *p* < 0.001) and less time to reach 100% granulation tissue within the wound (14.8 ± 7.3 vs. 44.6 ± 7.1 days, *p* < 0.001) [12]. The study also highlighted a reduction in number of inpatient days (15.7 vs. 29 days, *p* < 0.001) and a significant increase in healed wounds at 3 months (20 NPWT and 6 with saline dressings, *p* = 0.006) [12]. A larger multicentre study by Seidel et al. [13] conducted in 2020 included 345 patients, 171 in the intervention group (133 males and 38 females) and 174 in the SMWC cohort (134 males and 40 females) with mean age 67.6 years and 68.1 years respectively. The group identified no significant differences between intervention and control when comparing rate and time of wound closure [13]. A later study conducted in 2022 by Seidel et al. [14] involved 368 patients, 44 of whom had NPWT (29 males, 15 females mean age 66.5 ± 11 years) and 110 that had SMWC (84 males, 26 females, mean age 67.8 ± 10.4 years). NPWT was showed to lead to a significantly faster duration of treatment (82.8 ± 31.6 days vs. 98.8 ± 24.6 days, *p* = 0.001) [14].

Alamdari et al. [15] reported the outcomes of a comparative analysis between NPWT and silver sulfadiazine dressings in 60 patients, equally split between the 2 study groups (13 females and 17 males, mean age 70.3 ± 5.9 years in the NPWT group and 14 females and 16 males in the control group, mean age 71.8 ± 6.3 years). Following treatment, the study reported a significant reduction in wound surface area (*p* = 0.008) and depth (*p* = 0.002) in the intervention group compared with the control, as well as a lower rate of major (0 vs. five respectively) and equal number of minor amputations (7 in both groups) [15]. The group also noted a faster recovery and significantly lower disability period in the NPWT group (3 vs. 8 days, *p* = 0.01) [15]. Anjum et al. [16] has also followed a cohort of 40 patients, equally split between the intervention (12 males, 8 females, mean age 42.9 ± 9.3 years) and control group (16 males, 4 females, mean age 46.3 ± 9.3 years). While comparing VAC with saline‐soaked gauze therapy, Anjum et al. [16] noted a significantly reduced duration of wound healing in the intervention group (7.5 ± 2.8 days vs. 10.6 ± 5.5 days, *p* < 0.05).

For the comparison of VAC with conventional dressings, James et al. [17] included in their study a number of 54 patients with diabetic wounds, 27 in the intervention group out of which 16 males and 11 females and mean age 55.8 years, and 27 in the control group, 15 males and 12 females with a mean age of 52.9 years. The study showed a significant faster healing rate (3.8 vs. 22.5 days, *p* < 0.0001) and faster time to reach over 75% granulation tissue within the wound (23.3 vs. 32.1 days, *p* < 0.0001) in the VAC group [17]. Pain was significantly improved using NPWT when assessed using the Visual Analog Scale (VAS) score (*p* = 0.004), there were no statistically significant differences between the two groups regarding infection and bleeding within the wounds [17]. A lower pain score in the NPWT group was also identified in the study by Subbiah et al. [18] (*p* < 0.001), who compared 25 patients undergoing therapy using VAC (17 males, 8 females, mean age 54.8 ± 12.7 years) and 25 patients who received standard care with conventional dressings (20 males, 5 females, mean age 54.7 ± 8.7 years). The group also identified a significant increase in granulation tissue formation following VAC therapy (39.3 ± 2.5 mm vs. 34.4 ± 5.5 mm, *p* < 0.001) and significantly reduced period as inpatient (21.5 ± 2.2 days vs. 28.7 ± 3.6 days, *p* < 0.001) [18].

Srivastava et al. [19] provided a comparative analysis between 55 patients with diabetic wounds, 23 included in the NPWT intervention group (16 males, 7 females, mean age 37.3 ± 6.8 years) and 32 in the control (26 males, 6 females, mean age 36.7 ± 7.2 years). A significant percentage reduction in wound size in comparison with the baseline measurements was noted following NPWT dressings application at day 7 (*p* = 0.013), day 14 (*p* = 0.001) and day 21 (*p* = 0.029) and a significant reduction in the VAS pain score particularly at days 14 and 21 (*p* < 0.001 for both timepoints) when compared to the control [19]. Furthermore, no microbial growth was identified on a significantly higher number of wound in the intervention group on day 21 of the study (*p* = 0.008) [19].

#### 3.4.2 Efficacy and Sensitivity Analysis‐ Hospital Stay Duration and Rate of Amputations

**FIGURE 3:**
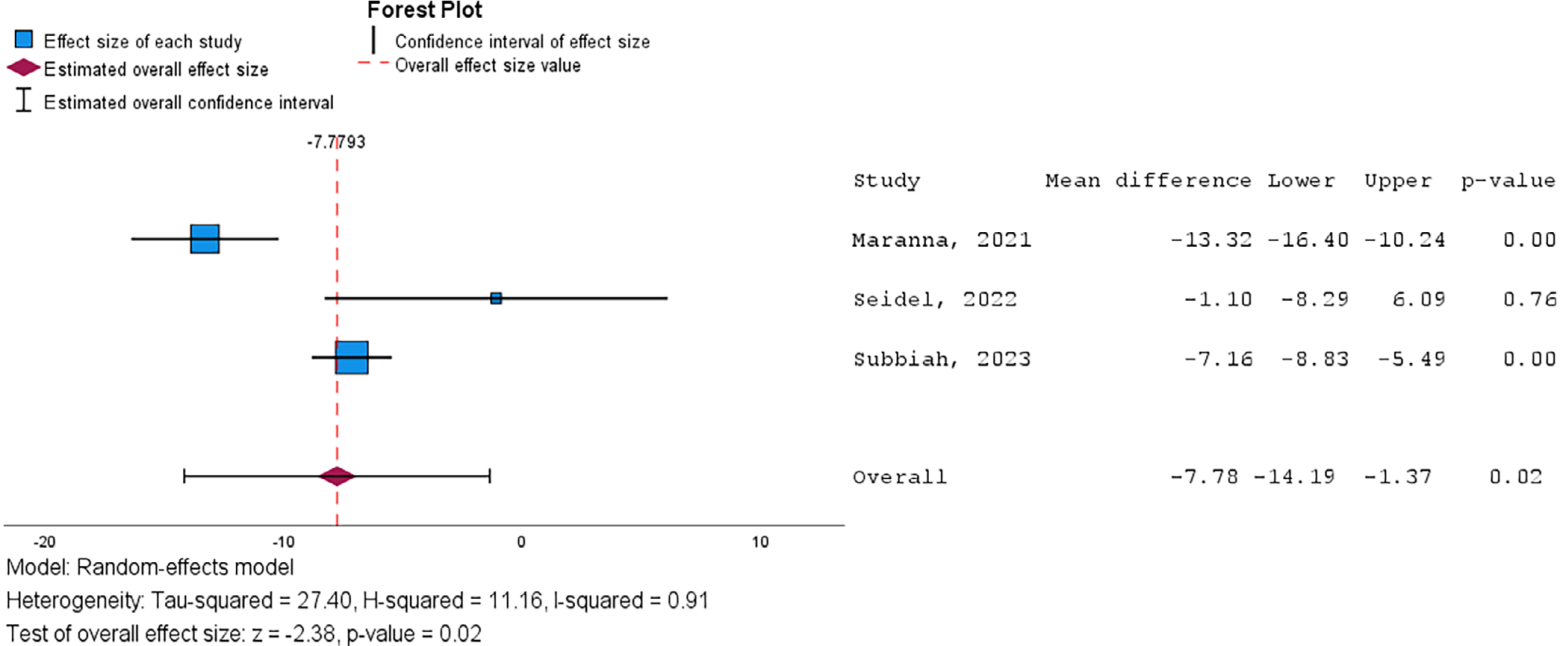
Forest plot of the effect sizes for mean duration of hospital stay analysis in NPWT vs. control.

All the studies included in the analysis indicated a reduction in the number of hospitalisation days (Table 3). The overall effect of the NPWT resulted from the meta‐analysis, showing a significant reduction (*p* = 0.017) in the number of hospitalisation days by 7.8 (95% CI: −14.2; −1.4) (Figure 3).

**TABLE 3:** Comparison of mean duration of hospital stay for patients using NPWT vs. control.

Study	Mean – hospitalisation duration/intervention	Mean – hospitalisation duration/control	StDev intervention	StDev control	N intervention	N control
Maranna et al. [12]	15.68	29	2.02	7.11	22	23
Seidel et al. [14]	14.6	15.7	17.4	21.7	44	110
Subbiah et al. [18]	21.52	28.68	2.23	3.64	25	25

There is a high level of heterogeneity among studies (*I* ^2^ = 91%), attributed to substantial differences between studies and the small number of studies included in the analysis. The cumulative effect regarding the difference in mean duration of hospital stay (intervention vs. control) is statistically significant (*Z* = −2.4, *p* = 0.017).

**FIGURE 4:**
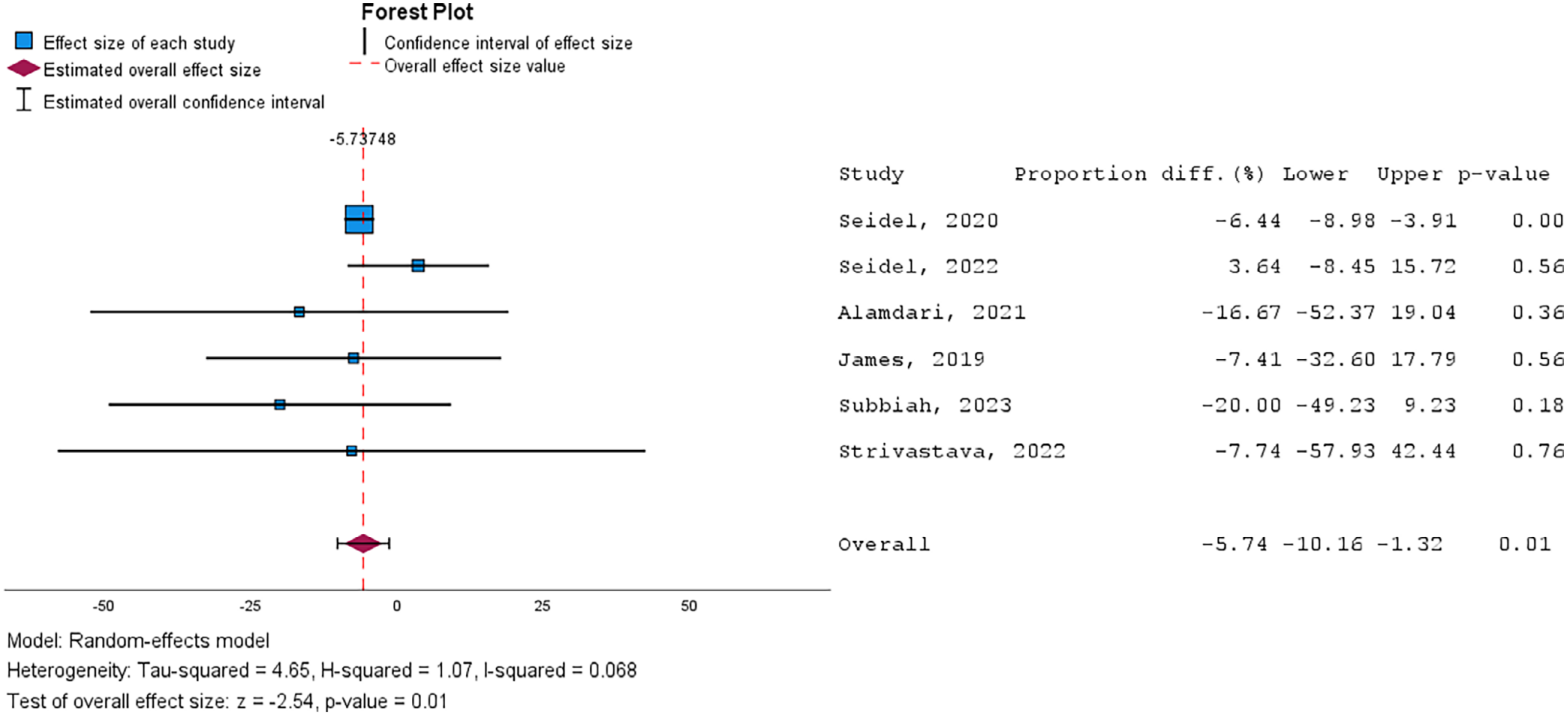
Forest plot of the effect sizes for the number of amputations following NPWT vs. control.

The studies that evaluated the number of major and minor amputations reported a lower count in the intervention group compared to the control group (Table 4). The result of the meta‐analysis indicated a significant reduction (*p* = 0.01) in the proportion of major and minor amputations by 5.7% (95% CI: −10.2 to −1.3) (Figure 4).

**TABLE 4:** Comparison of mean number of amputations for patients using NPWT vs. control.

Study	Number of major + minor amputations/intervention	Number of major + minor amputations/control	Number of major + minor amputations/intervention	Number of major + minor amputations/control	N intervention	N control
Seidel et al. [13]	45	57	26.32%	32.76%	171	174
Seidel et al. [14]	10	21	22.73%	19.09%	44	110
Alamdari et al. [15]	7	12	23.33%	40.00%	30	30
James et al. [17]	3	5	11.11%	18.52%	27	27
Subbiah et al. [18]	1	6	4.00%	24.00%	25	25
Strivastava et. al [19]	9	15	39.13%	46.88%	23	32

The heterogeneity of the studies included in the analysis was low (I^2^ = 6.8%), indicating that there are no significant differences between the results of the included studies.

The cumulative effect resulting from the analysis indicated a significant reduction in the number of major and minor amputations in the intervention group (overall effect size: −5.7; 95% CI: −10.2 to −1.3; *p* = 0.01).

### 3.5 Comparison of Various NPWT Strategies Through Clinical Studies

Al‐Sabbagh et al. [20] highlighted a significant increase in complete wound healing and a marked reduction in time to heal and rate of amputations results of t‐NPWT with an increase in negative pressure to −160 mmHg (high NPWT). Campitiello et al. [21] noted that wrapping the whole foot in the VAC dressing (NPWT+) improves the seal and brings margins closer together by stronger mechanical forces. Over the 3 weeks of the analysis, a significant reduction on healing time and higher rate of complete wound healing have been noted in the NPWT+ group compared to the traditional NPWT type of wound occlusion [21].

There have also been limited comparisons done between the different types of NPWT devices. While Kirsner et al. [22] identified that PICO, a single use portable NPWT device, helped increase the rate of wound closure compared to t‐NPWT, no significant differences in outcomes were observed in the study proposed by Davis et al. [23] between t‐NPWT and NPWT with irrigation when comparing proportion of healed wounds, wound closure, time to healing or hospital stay length.

### 3.6 Review of Bioscience Mechanisms of NPWT

#### 3.6.1 Understanding the Underlying Biomolecular Mechanisms of NPWT

Aside from a comparative analysis between NPWT and conventional dressings or a comparison of different types or application strategies of NPWT devices, the present systematic review has also identified some noteworthy studies aimed to delve into the potential biomolecular mechanisms influenced by NPWT in diabetic wound healing.

Wu et al. [24] demonstrated a reduction in NETs between NPWT and the control. The same study has also showed that NPWT can promote the phenotypic change of macrophages from M1 to M2, an important change in polarisation found in the normal wound healing pathways [33].

Regarding angiogenic factors, Hohendorff et al. [25] highlighted the downregulation of Ang2 post NPWT application could highlight a pro‐angiogenic change in behaviour in chronic wounds following negative pressure treatment.

When comparing the NPWT to standard care dressings, Wang et al. [26] noted a significant downregulation of the pro‐inflammatory TNF‐α, IL‐1β and IL‐6 and Tang et al. [29] identified significantly lower expression of inflammatory markers (white blood cell, neutrophil percentage, C‐reactive protein, procalcitonin) in the NPWT group after 1 week of therapy. Also, they showed there is a higher expression of tissue margins peroxiredoxin‐2 (t‐PRDX2) in the NPWT group. To contribute to the positive impact of NPWT in improving DFU healing, Liu et al. [27] demonstrated that these specialised dressings drastically lowered the levels of human microRNA 203 (hsa‐miR‐203).

#### 3.6.2 Genomics and Proteomics Analysis of Wound Tissues

Recent studies focusing on NPWT have also covered the genomic and proteomic analysis in DFU tissues and their effects compared to the standard care dressings. Therefore, Jia et al. [28] identified 33 upregulated and 3 downregulated proteins in granulation tissues following 1 week of NPWT, associated with inflammatory responses, antioxidation, complement and coagulation cascades, lipid metabolism and cytoskeleton. The study also noted the upregulation of PRDX2 in peripheral blood and granulation tissue, similarly to Tang et al. [29]. Moreover, Ludwig‐Slomczynska et al. [30] provided a genomic comparative analysis of DFU in the NPWT and standard therapy groups. This identified 426 differentially methylated regions after NPWT (unlike the control group), some which might contribute to inhibiting complement activation. Also, a further transcription factor analysis confirmed the presence of factors such as IRF6 and Hoxa3.

#### 3.6.3 In Vivo Studies

In vivo studies have also contributed to the understanding of the underlying effects of NPWT in wound healing. Thus, an in vivo study by Wu et al. [31] analysing the effects of NPWT on full‐thickness excisional dorsal skin wounds in diabetic mice demonstrated that NPWT downregulates a series of pro‐fibrotic factors such as Vimentin, α‐SMA and HSP47, and pro‐apoptotic factor Casoase‐3, while increasing the level of yes‐associated protein (YAP). The study also highlighted a significant increase in cellular proliferation and decrease in apoptosis within the 10‐days old wounds compared to the occlusive dressing control. Zhang et al. [32] has also analysed full‐thickness excisional wounds in diabetic mice and illustrated that NPWT triggers a significant upregulation of VEGF, p‐ERK and microRNA‐126 and results in a positive correlation between the levels of circulating microRNA‐126 and arteriolar and capillary density.

## 4 Discussion

It can be noted from the present systematic review that there is a paucity in the most up to date literature of studies that provide an in‐depth analysis of the NPWT effects in diabetic wound healing. Although some have tried to provide a comparative analysis of the clinical improvements observed with NPWT or conventional standard care dressings, the results are limited and firm conclusions regarding the superiority of one wound care strategy over the others is yet to be clearly stated. The limitations come not only in the observational studies on real‐life patient wound healing experience using the devices, but also in studies aiming to uncover some of the potential biological pathways that NPWT influence in diabetic wounds.

### 4.1 NPWT Vs. Standard Care Dressings

In the present systematic review, only 8 studies have compared t‐NPWt with the standard care dressings across multiple healthcare centres across the world. One notable result across 6 of the 8 studies presented was the lower number of minor and major amputations in the intervention group. However, only Alamdari et al. [15] and Subbiah et al. [18] noted a statistically significant decrease with NPWT when compared with the controls. Although a clear conclusion cannot be drawn from the results due to limitations such as large discrepancies in number of patients in study groups, no clear differentiation between minor and major amputations numbers in the analysis across multiple studies or the small number of studies that reported on this parameter, the decreasing trend in amputations number has been previously noted in the literature. A metanalysis by Zhang et al. [34] from 2014 identified that the NPWT groups had significantly less major amputation rates compared to the control, while the rate of minor amputations was not affected between the two groups.

Another parameter that has been analysed in multiple studies was the duration of hospital stay, which showed a significant decrease between the NPWT from controls in 2 out of the 3 studies (Maranna et al. [12] and Subbiah et al. [18]). This significant decrease in the hospitalisation period in favour of NPWT devices when compared to the controls has been also noted across a wider aetiology of wounds and mentioned previously in the literature in the systematic review and metanalysis of randomised control trials by Liu et al. [35]. This is a very important factor in the management of patients with challenging wounds both from a health economics perspective, but also a patient quality of life and overall experience throughout the wound healing process. Particularly in the case of chronic or difficult to treat wounds such as diabetic once, being able to improve the patient experience and reduce the time required to return to normal daily activities or provide care in an outpatient environment can make a significant difference in mental well‐being, reduce risk of hospital‐acquired infections or other complications and help in the overall wound management process.

### 4.2 NPWT Effects on Wound Healing

Interestingly, we identified only one study that has analysed the effects of different levels of negative pressure on wound healing. The results of Al‐Sabbagh et al. [20] suggesting a potential improvement in the rate and duration of complete wound healing as well as a reduction in amputation rates have been previously supported in the literature. Earlier studies add to the potential benefits of a higher pressure, Timmers et al. [36] showing that even at negative pressures of −300 mmHg a notable increase in blood flow within the wounds, which could be a contributor to improved wound healing. However, higher pressures might not always be advised, as it may potentially be a cause of pain [37] and, as there is a paucity of data analysing the effects of high pressure on diabetic wound healing, further clinical studies would be required to confirm the hypothesis.

### 4.3 Biological Pathways in Human and In Vivo Studies

Regarding studies analysing wound healing biomolecular pathways influenced by NPWT, Wu et al. [24] noted a decrease in NETs following NPWT. Although NETs can have a beneficial bactericidal role, in diabetic wounds the high NETs levels can be cytotoxic [38, 39] and result in tissue damage [24, 40]. Furthermore, timely phenotypic change of macrophages from M1 to M2 influenced by NPWT is an important step in normal wound healing pathways [33, 41]; chronic wounds generally present a delayed polarisation and prolonged high levels of M1 macrophages [42].

Hohendorff et al. [25] identified a downregulation of Ang2 post NPWT which highlights a pro‐angiogenic NPWT‐induced change in behaviour in chronic wounds. Previous studies have showed Ang2 role in angiogenesis fluctuates depending on VEGF expression, with pro‐angiogenic behaviours linked with VEGF expression, and high levels of anti‐angiogenic Ang2 influenced by VEGF inhibition such as in diabetic wounds [25, 43]. In vivo, Zhang et al. [32] also concluded that NPWT can lead to upregulation of VEGF and miRNA‐126. The latter is an important regulator of angiogenesis, expressed in healing diabetic wounds [44].

Inflammation also plays a significant role in diabetic wounds chronicity. The studies by Wang et al. [26] and Tang et al. [29] both noted downregulation of inflammatory factors following application of NPWT devices when compared to controls. Increased levels of inflammatory cytokines TNF‐α, IL‐1β and IL‐6 are often associated with a diabetic state and are known to perpetuate the inflammatory phase of healing, contributing to chronicity [45]. Particularly TNF‐α has been previously linked with delayed wound closure [45, 46] and degradation of connective tissue [45, 47], thus interfering with normal healing pathways.

Liu et al. [27] also demonstrated NPWT promotes downregulation of has‐miR‐203, a “skin‐specific” RNA molecule that inhibits expression of p63, essential gene in wound healing required for epithelial development [27, 48]. It has previously demonstrated that DFU exhibit upregulation of has‐miR‐203 [49] and can alter normal wound healing by inhibiting keratinocytes proliferation and migration [27, 50].

Wu et al. [31] In vivo study could offer a more in‐depth explanation of the NPWT role in wound scarring. The intervention groups noted a caspase‐3 downregulation, pro‐fibrotic factor involved in keloid scar formation [51], and YAP upregulation, essential factor in normal scaring [52]. It has been previously demonstrated that YAP signalling activation driven by mechanical forces enables engrailed‐1 (En1) expression; En1 deactivation due to YAP inhibition can lead to wound healing without fibrosis, which is regulated by a specialised subpopulation of fibroblasts named En1 linage‐negative fibroblast (ENFs) [52]. Although this would suggest the increase in YAP noted by Wu et al. [31] in NPWT‐treated wounds would lead to impaired healing and fibrotic changes, the study conversely noted the uncoupling of En1 and YAP. NPWT wounds were characterised by increased YAP expression, however a downregulation of En1 was noted, allowing a decreased fibrotic response within healing tissues [31]. This is overall believed to result due to inhibition of YAP nuclear sequestration, as supported by decreased Caspase‐3 and subsequent decrease in cleavage of a‐catenin, identified in NPWT‐treated wounds [31]. Therefore, this uncoupling of YAP and En1 in the mechano‐transduction pathway could be further investigated to explain the positive role of NPWT in promoting wound healing through appropriate tissue regeneration.

### 4.4 Limitations

A noteworthy limitation that needs considering is lack of standardisation for outcomes assessment. We noted a paucity of data starting from descriptive wounds information to outcomes of size reduction at particular time intervals; only 2 out of 8 studies provided such information, with clear baseline wounds' dimensions and measurements taken 14 days post‐treatment. Particularly noticeable when comparing NPWT with standard care, there is also a high variation in defining “healing”. While some considered “days to healing” the time of granulation tissue appearance, others noted other targets such as 75%, 95% or 100% granulation tissue formation. Considering the limited studies and small cohorts included, more uniform and comparable outcome measures would be beneficial and help support clear conclusion formulation.

Other factors such as time of complete healing, percentage reduction in size, number of complications or positive microbial wound cultures are also very scarcely presented—in our analysis, maximum of 2 studies contain the same outcome measures, making a direct comparison highly challenging.

Seidel et al. [13] has noted some several other important limitations, including issues in documentation, poor adherence to treatment plans and guidelines, recognising that results cannot create a clear conclusion on the efficiency of NPWT and should be further investigated.

## 5 Conclusions

The present systematic review covered some of most recent studies analysing the effects of NPWT in diabetic wounds. A total of 21 articles were reviewed, 8 of which assessed clinical efficiency of NPWT vs. standard care dressings, 5 RCTs looking into variations of NPWT including comparisons between t‐NPWT and single‐use devices or irrigation devices, changes in negative pressure levels, and dressing coverage strategy, 6 studies analysing biomolecular changes brought about by NPWT and 2 in vivo studies on full‐thickness excisional wounds in murine models. While there seems to be a consistent trend in favour of NPWT, results should be interpreted with caution until more robust studies with standardised reporting become available in specialised literature. Thus, definitive conclusions regarding the superiority of NPWT over standard care dressings in diabetic wound healing are yet to be defined. With some significant limitations mainly drawn by the lack of studies and extensive array of outcome measures, results of the mentioned studies cannot be compiled to strengthen the conclusions, weather favourable or not for NPWT devices.

To further improve the paucity of data in current literature, more uniform analysis timepoints or outcome measures such as a clear indication of start of healing should be reported in future studies. Also, considering the complexity of healing mechanisms and, particularly, of the underlying pathways that lead to chronicity, a more in‐depth analysis of biomolecular mechanisms of wound healing and effects of NPWT would be beneficial to assess its role in diabetic wound healing.

## Conflicts of Interest

The authors declare no conflicts of interest.

## Data Availability

Study is a systematic review and meta‐analsysis, not applicable for data sharing.
